# Success of airway management in out-of-hospital cardiac arrest using different devices - a prospective, single-center, observational study comparing professions

**DOI:** 10.1186/s13049-025-01422-2

**Published:** 2025-06-23

**Authors:** Nils Brenne, Niclas Brünjes, Dennis Rupp, Martin Christian Sassen, Andreas Jerrentrup, Hinnerk Wulf, Nils Heuser, Christian Volberg

**Affiliations:** 1 Department of Anesthesiology & Intensive Care Medicine, Faculty of Medicine, Philipps University of Marburg, 35043 Baldingerstraße, Marburg, Germany; 2 Department of Anesthesiology, Intensive Care Medicine and Pain Medicine, 58452 Marienhospital, Witten, Germany; 3 Medical Clinic I - Gastroenterology, General Internal Medicine, Diabetology and Geriatrics, Agaplesion Evangelisches Krankenhaus Mittelhessen, 35398 Gießen, Germany; 4 EMS Mittelhessen, German Red Cross, 35043 Marburg, Germany; 5 Department of Hazard Prevention, District of Marburg-Biedenkopf, Im Lichtenholz 60, 35043 Marburg, Germany; 6 Department of Emergency Medicine, DGD Diakonie-Hospital Wehrda, Hebronberg 5, 35041 Marburg, Germany; 7 Department of Emergency Medicine, Faculty of Medicine, Philipps University of Marburg, 35043 Marburg, Germany

**Keywords:** Out-of-hospital cardiac arrest, Endotracheal intubation, Video laryngoscopy, Airway management, Emergency medical services

## Abstract

**Background:**

Endotracheal intubation is the gold standard for airway management in out-of-hospital cardiopulmonary resuscitation (CPR) but requires practice, especially in difficult conditions. To facilitate this, video laryngoscopy (VL) is increasingly used. The extent to which it is actually used in practice by paramedics or emergency physicians (EPs) and its effectiveness remain unclear. This prospective observational study investigates these aspects.

**Methods:**

From January 2020 to June 2024, we surveyed emergency physicians and paramedics in a German county about out-of-hospital resuscitations. The questionnaire covered qualifications, airway devices, attempts, and complications. Data was analyzed descriptively and statistically. The significance level was set at alpha ≤ 0.05.

**Results:**

301 questionnaires were analyzed, with an overall first pass success (FPS) rate of 62.8%. No significant difference was found between direct laryngoscopy (DL) and video laryngoscopy (VL), though VL with McGrath performed worse than DL and VL with C-Mac. FPS rates did not differ significantly between emergency physicians and paramedics. Both achieved better results with their regularly used device. Paramedics used laryngeal masks more frequently (34% vs. 1.5%, *p* < 0.001). Among emergency physicians, anesthetists had the lowest FPS using DL (*p* < 0.001).

**Interpretation:**

The FPS rate did not differ between DL and VL but was low overall. This low rate, as well as the fact that emergency physicians and paramedics achieved comparable results in intubation, might indicate an increased need for training and further education in the area of airway management for emergency personnel. However, it can also be questioned to what extent ETI can be recommended at all, with supraglottic devices being an alternative. A possible benefit of more training can be seen in the higher success rates with the more frequently used devices in both professions. An uncertainty of paramedics regarding endotracheal intubation is also reflected in an more frequent use of laryngeal masks. The fact that internists who used VL more frequently had better FPS rates than anesthetists who intubated conventionally more often shows the potential of VL, especially under difficult out-of-hospital intubation conditions.

**Trial registration:**

The study was registered in the German Clinical Trials Register (DRKS ID: DRKS00021821, 12.06.2020).

**Supplementary Information:**

The online version contains supplementary material available at 10.1186/s13049-025-01422-2.

## Background

The annual incidence of out-of-hospital cardiac arrest (OHCA) is between 67 and 170 per 100.000 inhabitants in Europe. In 2023, approximately 55.000 people were resuscitated by the emergency medical services (EMS) in Germany, which corresponds to an incidence of approximately 65/100,000 in a total population of 84.6 million [1]. In addition to reperfusion, securing the airway and reoxygenation play a decisive role in the care of resuscitation patients. The need for prompt airway management with endotracheal intubation (ETI) is a recurring topic of discussion, as the number of attempts to secure the airway increases the rate of serious complications and fewer attempts are associated with better outcomes [2–9]. Repeated intubation attempts have been shown to be associated with an increase in the number of significant pauses in chest compressions [10].

The German EMS system is based on paramedics and emergency physicians (EP). In most cases, a paramedic is the first person to arrive at the scene of a cardiac arrest and is responsible for initial life-saving interventions, such as airway management. They are trained in ETI, but it is not known how often they actually perform this intervention. Emergency physicians are also trained in DL and ETI during their training, but depending on their specialization, they have limited opportunities to perform this procedure in their daily work, which may affect their experience with intubation. Depending on the organization responsible for the professional self-regulation of doctors (State chamber of physicians), evidence of 50 intubations is usually required to obtain the additional qualification of ‘emergency physician’, but regular use or refresher training is not required [5, 9, 11]. According to the 2019 German AWMF S1 guideline “Prehospital Airway Management”, an ETI should only be performed if the person intubating can demonstrate at least 100 guided intubations and regularly performs more than 10 ETI per year. At the same time, ETI remains the gold standard for airway protection, providing effective aspiration protection and potentially higher ventilatory pressures, as well as the ability to continue resuscitation with uninterrupted chest compressions and effective ventilations [7]. Given the existing controversy in resuscitation research regarding airway management, and the fact that the S1 guideline in Germany ultimately recommends against intubation for inexperienced users, and the ERC guideline recommends a 95% success rate of airway management within 2 attempts, which can only be achieved by such experienced users, it is interesting to see how airway management is performed during resuscitation in real life conditions [5, 12]. The aim of this study is to investigate how and by whom airway management is most performed in the field, whether success rates differ between emergency physicians and paramedics, and the extent to which the device used for laryngoscopy makes a difference to the success rates of the different professions.

## Methods

As recommended by the EQUATOR (Enhancing the Quality and Transparency of Health Research) network, the Strengthening the Reporting of Observational Studies in Epidemiology Statement (STROBE) guideline for observational studies was followed [13]. The completed checklist can be found in the Supplementary Appendix 1.

### Registration/ethics approval

The study was submitted to the institutional review board of the Philipps University of Marburg for ethical consideration with a positive vote (ref.: 134/19, 02.09.2019) and was registered in the German Clinical Trials Register (DRKS ID: DRKS00021821, 12.06.2020). Approval was also obtained from the concerning EMS agency and the Medical Director of the Rescue Service of the county.

### Data collection

From January 2020 to June 2024, emergency physicians and paramedics of one German EMS agency were surveyed. The agency records all OHCA resuscitations to evaluate it as part of internal quality management. In this way, the staff involved can receive objective feedback on the quality of resuscitation in terms of the quality of chest compressions, times and any deviations from the guidelines. A member of the EMS agency is responsible for processing the information from the CPR operations. It is then passed on to the staff involved in the resuscitation attempt as a form of objective feedback. Our digital questionnaire was sent out with this feedback and processed anonymously. All attempts of airway management by endotracheal intubation (ETI) or supraglottic airway management (SGA) during CPR were included. In addition to the number of attempts to secure the airway, the devices used (resuscitation bag, laryngoscope, various video laryngoscopes) and airway aids (Guedel tube, Wendl tube), as well as complicating factors (light or space conditions, patient mouth opening, vomiting, etc.), we collected personal data on qualifications and years of professional experience, as well as a personal assessment of one’s own skills in resuscitation. It was also possible to enter free text responses. If at least one complete questionnaire was submitted for an operation, we supplemented the dataset with information from the resuscitation feedback, identified by a given unique ID. For example, age, sex, initial heart rhythm, observed cardiovascular arrest, first responders or bystander resuscitation, and ROSC were included. By allowing any team member to provide feedback, we were able to track incorrect feedback or changes in responsibility within the team.

### Setting

All vehicles are equipped with Corpuls^®^ 3 (GS Elektromedizinische Geräte G. Stemple GmbH, Kaufering, Germany) which, in addition to defibrillation and monitoring (SpO_2_, ECG, (N)IBD, capnography), have a resuscitation feedback sensor (Corpuls^®^ Corpatch CPR Sensor) and can make their data available for evaluation via a digital interface. A C-MAC video laryngoscope (Karl Storz SE & Co. KG, Tuttlingen, Germany) with a hyperangulated blade (D-blade) is available in all rescue vehicles staffed with an emergency physician. McGrath video laryngoscopes (Medtronic GmbH, Meerbusch, Germany) with a hyperangulated blade (X-blade) are carried in selected ambulances. Laryngeal masks of the second generation LMA Supreme model and Ruesch endotracheal tubes with cuff and guide rod (both from Teleflex Medical GmbH, Fellbach, Germany) are used on all rescue vehicles. For conventional direct laryngoscopy, all vehicles are equipped with a conventional laryngoscope with McIntosh blades in various sizes. Paramedics and EPs are trained in DL and VL. Paramedics are assisted by EMTs, who are trained in bag-mask ventilation and the use of supraglottic airway management (SGA), such as the use of a laryngeal mask (LMA) [14].

During the first wave of the coronavirus pandemic, there was no further recruitment of patients between April 2020 and June 2020, following the instructions of the medical management and in consideration of the unclear impact on the daily work of the emergency services. The study was then able to continue without interruption, even during a renewed increase in infection rates. We did not observe any significant changes in the frequency and quality of resuscitation during this period and therefore do not see any bias in the study data.

### Statistical analysis

Data analysis and management were performed using Microsoft^®^ Excel Version 16.94 (25020927) and SPSS^®^ Statistics version 27 (IBM Corp., Armonk, NY, USA). Mean with standard deviation and median with interquartile range (IQR) were calculated as measures of central tendency. Several qualitative characteristics were tested for dependence using Chi^2^- and t-tests. The significance level was set at alpha ≤ 0.05. The Bonferroni correction was applied to adjust for multiple testing.

## Results

### Participants and descriptive data

During the observation period, a total of 791 resuscitations were performed. Of these, the rescue personnel of 534 resuscitation attempts (67.5%) were asked to participate in the study. Exclusion criteria for not receiving a questionnaire were airway management prior to resuscitation and airway management after return of spontaneous circulation (ROSC). A total of 477 (89.3%) responses were received, of which 301 (63.1%) resuscitation attempts on adult patients were included in the study. The most common reason for exclusion was incomplete and therefore unusable questionnaires (e.g. missing device, which made further meaningful analysis impossible). The patients were 68.8 ± 14.6 years old on overage. 33.2% (*n* = 100) of the patients have been admitted to the destination hospital with a ROSC.

Table 1 describes the demographics of all patients included in the study. Baseline characteristics such as age and gender were recorded, as well as variables related to the resuscitation itself, such as initial heart rhythm and duration of resuscitation. Due to the asymmetric distribution and outliers in both directions, the median and interquartile range were used to describe the duration. The variables are presented as total numbers and as a percentage of the total sample size. EMS personnel were asked about their qualifications and work experience. Experience is presented as mean ± standard deviation (SD).

**Table 1:** Demographic data of patients and rescue personnel

**Patients:**	
**Total [n|**	301
**Age [y]**	68.8 ± 14.6
**Gender [n]**	
♂	204 (68.0%)
♀	96 (32.0%)
**Bystander-CPR [n]**	
Yes	167 (55.5%)
No	134 (44.5%)
**Inital Cardiac Rhythm [n]**	
Shockable	76 (25.3%)
*Ventricular Fibrillation*	75 (24.9%)
*Pulseless Ventricular Tachycardia*	1 (0.3%)
Non-Shockable	225 (74.7%)
*Asystole*	146 (48.5%)
*Pulseless Electrical Activity*	79 (26.3%)
**Median Duration of Resuscitation [min]**	21 (14–30)
**ROSC at hospital admission [n]**	
Yes	100 (33.2%)
No	201 (66.8%)
**Rescue Personnel:**	
**Emergency Physicians [n]**	197 (65.4%)
Professional Experience [y]	10.7 ± 7.6
*Anesthesiology [n]*	114 (58.8%)
*Internal Medicine [n]*	41 (21.1%)
*Orthopedic Trauma Surgery [n]*	20 (10.3%)
**Paramedics /EMT‘s [n]**	95 (31.6%) / 9 (3.0%)
Professional Experience [y]	8.0 ± 8.1

### Main results

#### First pass success

**Fig. 1:**
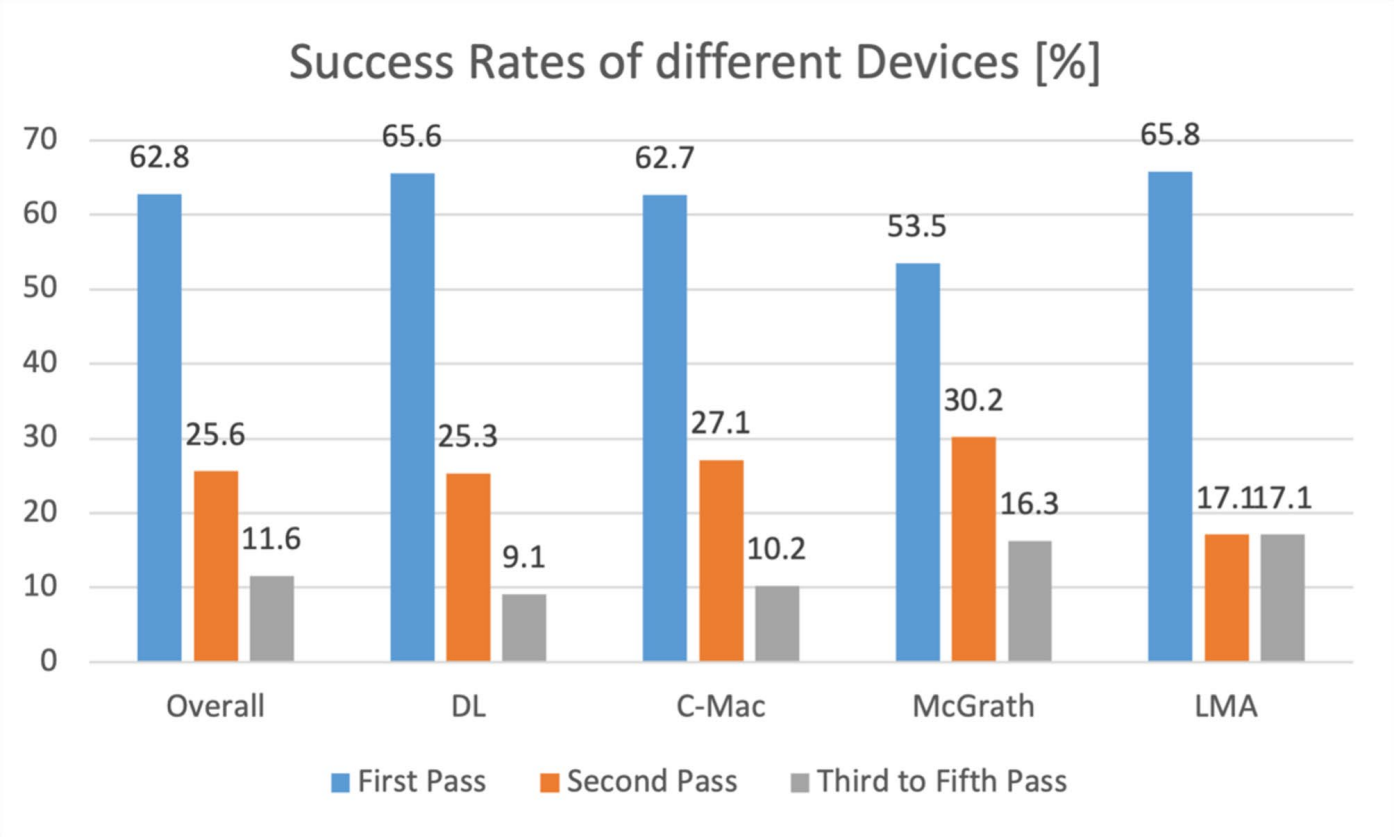
First to fifth pass success rates overall and for different devices

The first pass success rate overall (ETI + SGA) was 62.8% (*n* = 189). The FPS of ETI was 62.3% (*n* = 162). In all of the patients, the airway could be successfully secured with either ETI or SGA. In 3 of the cases, the airway was successfully secured at the 5th attempt. In this context, a repeat attempt meant a repeat laryngoscopy for endotracheal intubation or the re-insertion of a laryngeal mask. Re-positioning the tube did not count as a new attempt. Figure 1 shows the success rates of airway management overall and with the different devices analysed. Direct conventional laryngoscopy was compared with C-Mac and McGrath video laryngoscopy and supraglottic airway management with a laryngeal mask. The percentage of first and second pass success rates are presented. In the remaining patients, the airway was successfully secured after a maximum of 5 attempts. No patient had a misplaced tube or other unsuccessful airway management.

#### FPS using different devices

**Fig. 2:**
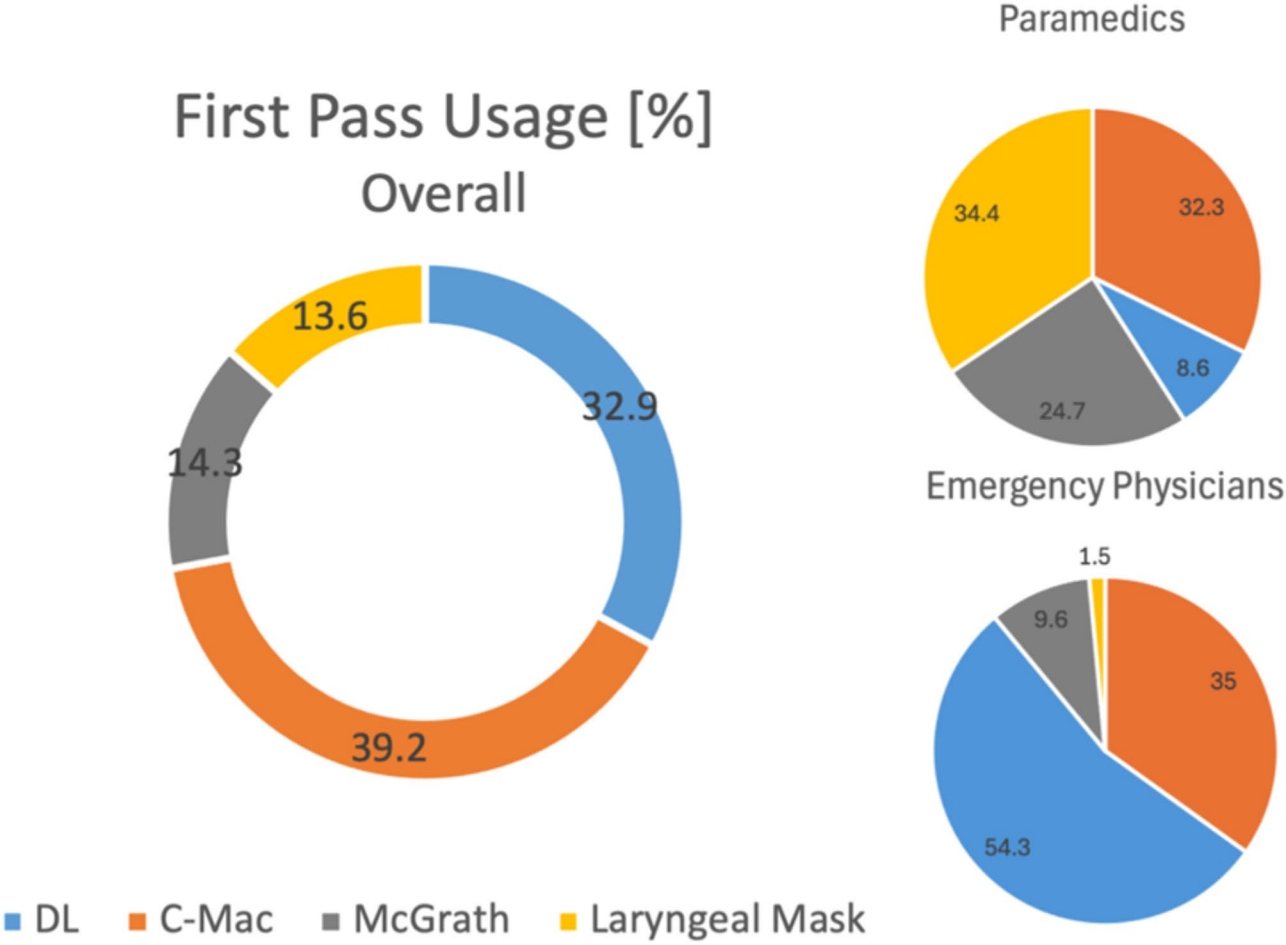
First pass usage overall and by different professions

We analyzed whether there was a difference in first pass success between different devices. Endotracheal intubation with a conventional direct Laryngoscopy (DL) was compared with video-assisted indirect Laryngoscopy (C-Mac/McGrath). In 65.4% (*n* = 193) of the cases airway management was performed by an emergency physician. In 30.9% (*n* = 93) airway management was performed by a paramedic. The most used device by EP’s was C-Mac, followed by direct laryngoscopy. Paramedics were significantly more likely to use a laryngeal mask (*p* < 0.001). The proportion of first pass usage for different devices is shown in Fig. 2. The results of first pass success rates are shown in Table 2.

**Table 2:** First pass success of different devices

	DL	C-Mac	McGrath	Laryngeal Mask
n (total)	99 (32.9%)	118 (39.2%)	43 (14.3%)	41 (13.6%)
First Pass Success total [n]	65 (65.7%)	74 (62.7%)	23 (53.5%)	27 (65.9%)
				***p*** **-value**
DL vs. VL				0.30
DL vs. C-Mac				0.65
DL vs. McGrath				0.17
C-Mac vs. McGrath				0.29

Figure 2 shows the percentage of the use of the different devices analysed overall and by different professions. Emergency physicians were compared with paramedics. Paramedics were more likely to use the McGrath video laryngoscope due to availability. Paramedics were also significantly more likely to use supraglottic airway management in the form of a laryngeal mask, whereas emergency physicians were more likely to use conventional direct laryngoscopy.

Table 2 describes the main results of the comparison between the different devices. The Bonferroni correction was applied to adjust for multiple testing. The results are presented as totals and percentages of successful first attempts of airway management. The results were compared and tested for dependence using the Chi^2^- test. No significant differences could be analysed.

#### FPS of different professions

Furthermore, we observed if there was a difference in the first pass success rates between emergency physicians and rescue service personnel and between emergency physicians with different specializations.

Table 3 describes the main results comparing the different professions in the use of the different airway management devices. The results are presented as totals and percentages. The Chi^2^- test was used to test for differences and dependence. No significant differences were found in the first pass success of the different professions. Analysis of the proportion of use of the different devices showed that paramedics were more likely to opt for supraglottic airway management, whereas emergency physicians were more likely to opt for ETI. Anaesthetists were more likely to use conventional direct laryngoscopy, while internists were more likely to use a video laryngoscope.

**Table 3:** First pass success of DL/VL of emergency physicians compared to rescue service personnel and of emergency physicians with different specializations using different devices

	First Pass Success [%]			*p*-value
**Emergency Physician**	63.4 (*n* = 123)			0.53
**Rescue Service Personnel**	59.1 (*n* = 39)			
**DL**				
Emergency Physician	64.7 (*n* = 44)			0.77
Rescue Service Personnel	67.7 (*n* = 21)			
**VL**				
Emergency Physician	62.7 (*n* = 79)			0.23
Rescue Service Personnel	51.4% (*n* = 18)			
**C-Mac**				
Emergency Physician	64.5 (*n* = 69)			0.17
Rescue Service Personnel	45.5 (*n* = 5)			
**McGrath**				
Emergency Physician	52.6 (*n* = 10)			0.18
Rescue Service Personnel	54.2 (*n* = 13)			
**Anesthesiology**	54.4 (*n* = 62)			0.06
**Internal Medicine**	75.6 (*n* = 31)			
**Orthopedic Trauma Surgery**	60.0 (*n* = 12)			
	**Percentage of Usage**	***p*** **-value**	**First Pass Success [%]**	***p*** **-value**
**VL**				
Anesthesiology	57.9 (*n* = 66)	0.32	51.5 (*n* = 34)	0.01
Internal Medicine	82.9 (*n* = 34)		82.4 (*n* = 28)	
Orthopedic Trauma Surgery	84 (*n* = 17)		58.8 (*n* = 10)	
**DL**				
Anesthesiology	39.5 (*n* = 45)	< 0.001	57.8 (*n* = 26)	0.71
Internal Medicine	17.1 (*n* = 7)		42.9 (*n* = 3)	
Orthopedic Trauma Surgery	15.0 (*n* = 3)		66.7 (*n* = 2)	
**C-Mac**				
Anesthesiology	45.6 (*n* = 52)	< 0.001	51.9 (*n* = 27)	0.01
Internal Medicine	75.6 (*n* = 31)		83.9 (*n* = 26)	
Orthopedic Trauma Surgery	85.0 (*n* = 17)		58.8 (*n* = 10)	
**McGrath**				
Anesthesiology	12.3 (*n* = 14)	0.19	50 (*n* = 7)	0.60
Internal Medicine	7.3 (*n* = 3)		(66.7 (*n* = 2)	
Orthopedic Trauma Surgery	0.0 (*n* = 0)		0 (*n* = 0)	

### Additional analysis

#### Complications

**Fig. 3:**
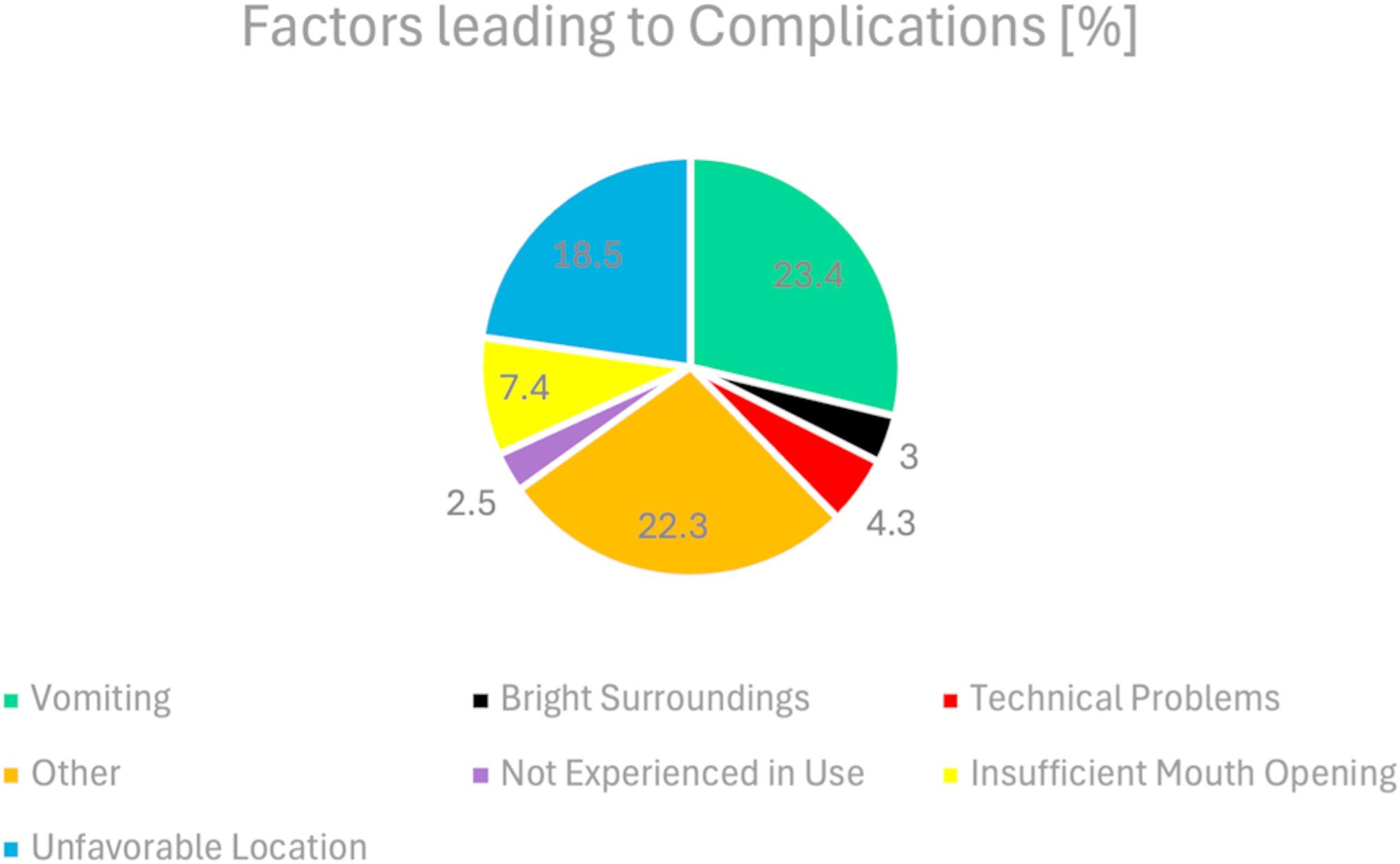
Factors leading to complications

The questionnaire also asked about complications during airway management. Multiple responses were possible. No complication was reported in 18.5% (*n* = 73) of the cases. The most frequent complications were vomiting and unfavorable spatial conditions. Figure 3 shows the factors that led to complications during airway management in out-of-hospital cardiac arrest. Multiple answers were possible. The most common answers are shown in the graph. The rest fall into the category ‘other’.

## Discussion

In out-of-hospital CPR, it is essential to secure the airway as early and fast as possible in order to provide continuous chest compressions and adequate oxygenation [15]. This poses a significant challenge for emergency personnel, whether emergency physicians or paramedics, as optimal intubation conditions cannot be expected in pre-hospital emergency situations. Patients are rarely on an empty stomach, and the scene itself can be a problem, for example due to cramped conditions or poor lighting. In our study, 81.5% (*n* = 228) of cases experienced problems during the first intubation attempt (Fig. 3). Vomiting and poor room conditions were the two most common problems leading to complications in airway management. These and similar factors are often difficult to control in the acute setting. However, one factor that can be influenced is the choice of device used to secure the airway [16]. Recently, video laryngoscopes have been increasingly used in out-of-hospital practice. Previous studies have shown that these might not generally increase the rate of first pass success, but can be a helpful tool for less experienced personnel and have a positive impact on patient outcome [17–22]. A recent meta-analysis by Araújo et al. even showed, that VL is a safer option for successful intubation at first attempt compared to DL in critically ill patients in general, and may reduce the risk of intubation failure [23]. In contrast to this, other studies did not observe better first pass success rates [9, 24, 25]. Another factor in the success of airway management is undoubtedly who is performing the procedure and how experienced they are. A difference between physicians and non-physicians in this matter has been observed [26, 27]. This poses a problem because paramedics are often responsible for primary airway management and failed initial intubation attempts are an independent risk factor for a decreased effectiveness of ACLS [28]. Furthermore an increased number of intubation attempts is associated with worse neurological outcome and more adverse events [2, 4]. In our prospective study, the factors of “device” and “user” were considered together and analyzed for associations.

### FPS rates using different devices

The first pass success of overall airway management was 62.8%. The airway has successfully been secured in all 301 patients and no misplacements have been observed. Previous studies have reported rates of misplaced tubes in the single digits for pre-hospital endotracheal intubation [29–31]. An older study from the USA even reported failure rates as high as 25% among paramedics [32]. In this study, we did not observe any such trends. Regarding video laryngoscopy, the overall difference of FPS rates between direct conventional laryngoscopy (DL) and indirect video-laryngoscopy (C-Mac/McGrath) did not show significance, yet the rate of successful first intubation attempts was higher with C-Mac and DL than with McGrath (63%/66% vs. 54%, *p* = 0.29/0.17, Table 2). Overall, emergency physicians achieved slightly higher FPS rates than paramedics in endotracheal intubation (63.4% vs. 59.1%, *p* = 0.53, Table 3). This association did not turn out to be significant. Paramedics were even able to achieve slightly better results in DL (67.7% vs. 64.7%, *p* = 0.77, Table 3). There also appeared to be an association between higher FPS rates and more frequent use of the device, with paramedics showing better rates with the McGrath VL and emergency physicians with the C-Mac (Table 3). This is not surprising given that the McGrath is carried on non-physician-staffed ambulances and the C-Mac is carried only on physician-staffed vehicles, suggesting that they are used more frequently by the respective professions.

### Differences between paramedics and emergency physicians

The fact, that paramedics showed a significantly higher use of supraglottic airway management highlights a possible uncertainty about endotracheal intubation and laryngoscopy. While emergency physicians almost never used it (1.5%), more than a third (34.4%) of all paramedics chose the laryngeal mask as the first tool to secure the airway (Fig. 2). Given that there are studies reporting that patients benefit from intubation compared to SGA in terms of ROSC, survival and neurological outcome, one conclusion could be that there is a need for greater inclusion of airway management in paramedic education and training [33–37]. The effect of such measures can be seen in non-emergency physician-based EMS systems such as Australia, Canada and the United States of America, where paramedics achieve higher FPS rates the more experience they have and those rates can be achieved by intensive training [38–40]. They even result in an improved survival [41]. However, there are also studies that show different results, such as the AIRWAYS 2 randomised clinical trial, which found no difference in functional outcome between SGA and ETI, nor in the risk of regurgitation and aspiration [42]. Another study even reported better 72-hour survival with a Laryngeal Tube (LT) than with ETI, and one study concluded that early airway management may be more important than the device used [43, 44]. When discussing the advantages of SGA over ETI, one might expect higher FPS rates with SGA given the less complicated insertion handling. In this study, the FPS rate of LMA did not differ from that of ETI. This observation could be due to a variety of reasons, such as the difficult conditions during OHCA or the fact that paramedics, who used SGA more often, are also less experienced in the use of a SGA compared to emergency physicians. However, it is also possible that the device itself is the cause of these low rates, and that other devices for SGA may give better results. For example, studies have shown that the I-Gel laryngeal mask is easier to use and quicker to insert than conventional laryngeal masks [45, 46].

### Differences between emergency physicians of different specialization

We were also able to show that among emergency physicians, anesthetists had the lowest overall FPS rate compared with internists and orthopedic trauma surgeons (Table 3). Although this trend was not significant and may be related to the differences in the number of intubations of the different specialties, it is interesting to note that anesthetists were significantly more likely to intubate conventionally and not use a video laryngoscope. In comparison, internists and orthopedic trauma surgeons used C-Mac significantly more often and internists also showed significantly better FPS rates using VL/C-Mac (*p* = 0.01/0.01; Table 3). The extent to which the lower FPS rate of anesthetists is related to the higher rate of conventional intubation is questionable. However, it is noteworthy because it is conceivable that anesthetists, due to their daily work in the hospital where they routinely perform ETI using DL, may also feel more confident in this procedure in an out-of-hospital setting than other specialists and therefore be more likely to perform conventional laryngoscopy despite the poor intubation conditions. The lower FPS rates of physicians using DL and the fact that the overall FPS rates of physicians did not differ from those of paramedics significantly indicates that it may be worthwhile to focus on training in the use of video laryngoscopy in this profession as well. This is supported by the fact that internists, who used C-Mac more frequently than anesthetists, also achieved significantly better FPS rates with this device. However, given the low FPS rates, regardless of the device, and the recommendations of the ERC guidelines in this regard, it is also worth considering avoiding endotracheal intubation by inexperienced personnel and relying solely on supraglottic airway devices. In out-of-hospital practice, where poor intubation conditions are common, everyone, regardless of their qualifications, should use every useful tool to avoid multiple attempts of airway management. This applies not only to video laryngoscopy, but also to the use of supraglottic airway devices, which do not offer reliable protection against aspiration, but may compensate for this disadvantage by faster and safer insertion and shorter interruption of chest compressions.

### Limitations

Although this study’s prospective design is a strength, it can only show associations. The causality of associations or possible differences can only be assumed. The data examined were collected from one German EMS response area, so our results may differ from other areas in Germany or other countries. As mentioned in the background section, the German EMS is based on paramedics and emergency physicians who come together in the rendezvous system in life threatening emergencies. It is conceivable that other outcomes could be measured in a differently structured emergency physician system. The same applies to non-emergency physician-based systems. Concerning the investigated devices a limitation is, that not every device was available on every rescue vehicle, so this could have influenced the number of attempts with the respective device. While C-Mac was available on all vehicles with an emergency physician, McGrath was available on selected ambulances staffed by paramedics. Only laryngeal masks and conventional laryngoscopy was available on all rescue vehicles. Also, not all employees were trained on every device. The data is derived from questionnaires. We assume that these were completed as conscientiously as possible. Possible misrepresentations (e.g. due to response bias) cannot be ruled out. Furthermore, not all of the survey questions were answered by every participant, meaning that there were some deviations from the total number of the participants concerning single aspects. A possible risk of recall bias cannot be excluded, as the time between the resuscitation and the completion of the questionnaire was not taken into account. The questionnaire included questions about complications during airway management. To adjust for selection bias, all EMS personnel involved in a resuscitation attempt received the questionnaire. If more than one questionnaire was completed, they were checked for congruence. Potential participation or response bias cannot be completely excluded.

## Conclusion

Although this study found no significant difference in the FPS rates between direct laryngoscopy and video laryngoscopy, the more frequent use of conventional laryngoscopy was associated with a lower FPS rate among anesthetists. Both, emergency physicians and paramedics had better FPS rates with the more frequently used video laryngoscope. Paramedics were more likely not to attempt endotracheal intubation and to use supraglottic airway devices. Given the importance of securing the airway during resuscitation attempts, to ensure adequate ventilation, more emphasis should be placed on airway management in the education and training of emergency personnel to optimize the process of out-of-hospital cardiopulmonary resuscitation and provide a better outcome for patients. As patients benefit from a shorter duration of resuscitation and repeated intubation attempts are associated with worse outcomes, tools such as video laryngoscopy and supraglottic airway devices should be used to improve the already difficult conditions for airway management and enable success on the first attempt.

## Electronic Supplementary Material

Below is the link to the electronic supplementary material.

Supplementary Material 1: Appendix

## Data Availability

The datasets used and analysed in the current study are available from the corresponding author on reasonable request.

## Abbreviations

ALS: Advanced Life Support
AWMF: Arbeitsgemeinschaft der Wissenschaftlichen Medizinischen Fachgesellschaften (Working Group of the Scientific Medical Societies)
CPR: Cardiopulmonary Resuscitation
DL: Direct Laryngoscopy
EMS: Emergency Medical Service
EMT: Emergency Medical Technician
EP: Emergency Physician
ERC: European Resuscitation Council
ETI: Endotracheal Intubation
FPS: First Pass Success
LMA: Laryngeal Mask
LT: Laryngeal Tube
OHCA: Out-Of-Hospital Cardiac Arrest
ROSC: Return Of Spontaneous Circulation
SGA: Supraglottic Airway
VL: Video Laryngoscopy
